# Combined Amplicon Pyrosequencing Assays Reveal Presence of the Apicomplexan “type-N” (cf. *Gemmocystis cylindrus*) and *Chromera* *velia* on the Great Barrier Reef, Australia

**DOI:** 10.1371/journal.pone.0076095

**Published:** 2013-09-30

**Authors:** Jan Šlapeta, Marjorie C. Linares

**Affiliations:** 1 Faculty of Veterinary Science, University of Sydney, Sydney, New South Wales, Australia; 2 School of Molecular Bioscience, University of Sydney, Sydney, New South Wales, Australia; University of Guelph, Canada

## Abstract

**Background:**

The coral is predominantly composed of the metabolically dependent coral host and the photosynthetic dinoflagellate *Symbiodinium* sp. The system as a whole interacts with symbiotic eukaryotes, bacteria and viruses. *Gemmocystiscylindrus* (cf. “type-N” symbiont) belonging to the obligatory parasitic phylum Apicomplexa (Alveolata) is ubiquitous in the Caribbean coral, but its presence in the Great Barrier Reef coral has yet to be documented. Approaches allowing identification of the healthy community from the pathogenic or saprobic organisms are needed for sustainable coral reef monitoring.

**Methods & Principal Findings:**

We investigated the diversity of eukaryotes associated with a common reef-building corals from the southern Great Barrier Reef. We used three tag encoded 454 amplicon pyrosequencing assays targeting eukaryote small-subunit rRNA gene to demonstrate the presence of the apicomplexan type-N and a photosynthetic sister species to Apicomplexa - *Chromera* *velia* . Amplicon pyrosequencing revealed presence of the small-subunit rRNA genes of known eukaryotic pathogens ( *Cryptosporidium* and *Cryptococcus* ). We therefore conducted bacterial tag encoded amplicon pyrosequencing assay for small-subunit rRNA gene to support effluent exposure of the coral. Bacteria of faecal origin (Enterobacteriales) formed 41% of total sequences in contrast to 0-2% of the coral-associated bacterial communities with and without *C* *. velia* , respectively.

**Significance:**

This is the first time apicomplexan type-N has been detected in the Great Barrier Reef. Eukaryote tag encoded amplicon pyrosequencing assays demonstrate presence of apicomplexan type-N and *C. Velia* in total coral DNA. The data highlight the need for combined approaches for eukaryotic diversity studies coupled with bacterial community assessment to achieve a more realistic goals of defining the holobiont community and assessing coral disease. With increasing evidence of Apicomplexa in coral reef environments, it is important not only to understand the evolution of these organisms but also identify their potential as pathogens.

## Introduction

The coral reef is traditionally defined by the cnidarian-*Symbiodinium* symbiosis [1,2]. However, the associated community of microorganisms such as bacteria, archaea, viruses, fungi, endolithic algae, and other eukaryotes are gaining significance in health and disease of tropical coral reefs [3,4].

Apicomplexa has been described as a key eukaryotic phylum in essentially all living vertebrate and invertebrate groups, including reef building coral. It comprises highly successful parasites which cause diseases of human (e.g. malaria, *Cryptosporidiosis* ) and veterinary (e.g. coccidiosis) importance and represent a substantial global economic and healthcare burden [5]. It even appears that modern apicomplexan parasites likely started out as mutualistic symbionts of coral before turning to parasitism [6]. The closest extant group to Apicomplexa (represented by photosynthetic alga - *Chromera* *velia* ) was isolated and cultured from coral species of the southern Great Barrier Reef [6]. Yet, very little is known about the role of *C* *. velia* in the coral holobiont and techniques to routinely test for *C* *. velia* are not available. A true apicomplexan parasite of coral was discovered in the Caribbean in the early 1980s. It was found in mesenterial filaments of several coral species and named *Gemmocystiscylindrus* [7]. Whether *G* *. cylindrus* is capable of causing disease is yet to be demonstrated, nevertheless it has been suggested as a contributing factor in White plague lesions of Caribbean coral [8]. In addition, sequences dubbed “apicomplexan type-N” detected in the Caribbean coral have been speculated to represent *G* *. cylindrus* [9].

The aim of this study was to test the ability of available 454 amplicon pyrosequencing methods to detect *C* *. velia* and the apicomplexan type-N in the coral holobionts of the southern Great Barrier Reef in Australia. We explored the feasibility of using the 454 amplicon pyrosequencing assays to detect low abundance eukaryotes and compared overlapping PCR primer pairs (V1-V4 region of the small subunit rRNA gene) to investigate the community composition within total coral holobiont DNA. In combination, the assays demonstrated presence of both *C* *. velia* as well as apicomplexan type-N.

## Material and Methods

### Coral DNA samples

Coral DNA samples were donated for this study. Coral samples were originally collected under a permit provided by the Great Barrier Reef Marine Park Authority. The samples were collected at reef locations surrounding One Tree Island (23°25’ S 151°55’ E) and Heron Island (23°25’ S 151°55’ E), both located in the Capricorn Bunker group of the southern Great Barrier Reef. Colonies of apparently healthy coral species *Acroporapalifera* 42a, *Montipora* *digitata* 60a, *Porites* *cylindrica* 66-2 from Heron Island (intertidal reef flats, July 2001) and *Seriatopora* *hystrix* 176-2 from the One Tree Island (notch at 10-13 m depth, July 2001) (Stat, M. 2006. The evolutionary history of coral-*Symbiodinium* assemblages: diversity and temporal stability. PhD thesis, The University of Sydney). Tissue and mucus from coral samples consisting of the outer 3–4 cm of a branch tip were removed from the skeleton with directed air jets of high pressure air supplied using SCUBA, and slurry was preserved in salt-saturated dimethyl sulfoxide (DMSO) conservation buffer as previously described [2]. DNA was extracted from blasted coral slurry using proteinase K and standard CTAB protocol [2]. The DNA was stored as aliquots at -80C.

### Eukaryote and Bacterial Tag Encoded FLX-Titanium Amplicon Pyrosequencing

The DNA extractions were submitted for eukaryotic and bacterial tag encoded FLX-Titanium amplicon pyrosequencing (TEFAP) using Titanium chemistry on 454 GS FLX System (Roche) at the Research and Testing Laboratory (RTL, Lubbock, TX) followed standard protocols [10]. Following sequencing, all failed sequence reads, low quality sequence ends, and tags were removed and sequences were depleted of chimeras at RTL [10] as described at http://www.researchandtesting.com/docs/Data_Analysis_Methodology.pdf.

**Figure 1:**
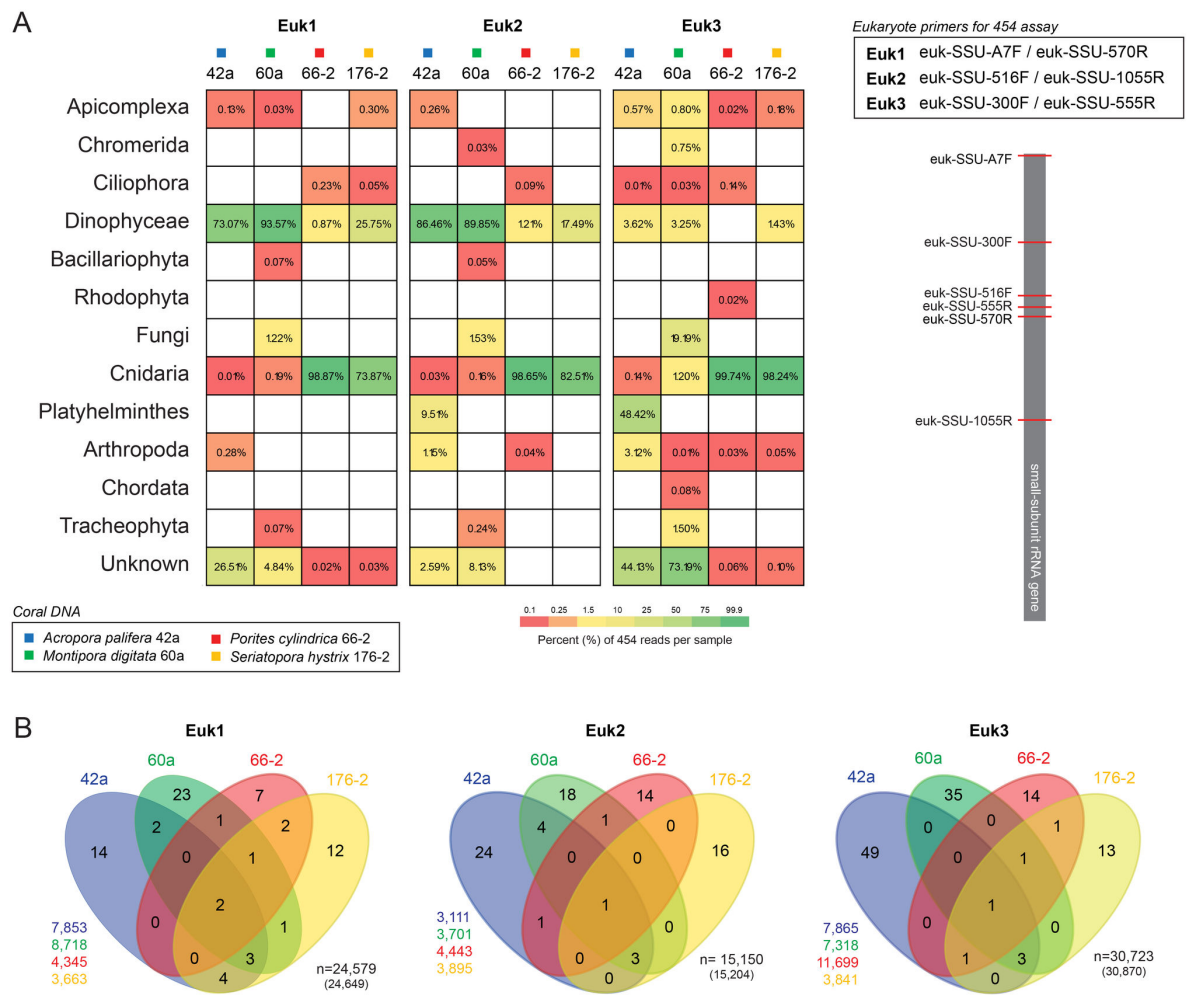
Comparative distribution of eukaryotic sequences in Great Barrier Reef coral holobionts. (A) Abundance of sequences belonging taxonomic groups of Eukaryotes in corals using three tag encoded 454 amplicon pyrosequencing assays targeting eukaryote small-subunit rRNA gene assays (Euk1, Euk2, Euk3). Relative position of the universal primer pairs utilised Euk1-3 assays is schematically shown on a complete small-subunit rRNA gene (inset right). (B) Venn diagram of sequence 97% sequence identity clusters shared between corals according to Euk1-3 assays. Number of sequences used to calculate Venn diagram within 97% clusters (>1 sequence) obtained using Euk1-3 assay and DNA indicated on the left (colour coded according to coral DNA). Total number of sequences recovered for each Euk1-3 (number in brackets on the right) and number sequences represented by >1 sequence (n). Coral species *Acroporapalifera* 42a, *Montipora* *digitata* 60a, *Porites* *cylindrica* 66-2 from Heron Island and *Seriatopora* *hystrix* 176-2 from the One Tree Island.

Three overlapping small subunit rRNA gene primer pairs (V1-V4 region of the small subunit rRNA gene) were used for amplifying ~250-600 bp region (Figure 1); Assay Euk1 (>400 bp) euk-SSU-A7F (5’-AAC CTG GTT GAT CCT GCC AGT) and euk-SSU-570R (5’-GCT ATT GGA GCT GGA ATT AC), Euk2 (>400 bp) euk-SSU-516F (5’-GGA GGG CAA GTC TGG T) and euk-SSU-1055R (5’-CGG CCA TGC ACC ACC), and Euk3 (<400 bp) euk-SSU-300F (5’- AGG GTT CGA TTC CGG AG) and euk-SSU-555R (5’-GCT GCT GGC ACC AGA CT). The 16S universal eubacterial primers 16S-28F (5’-TTT GAT CNT GGC TCA G) and 16S-519R (5’-GWN TTA CNG CGG CKG CTG) were used (assay Bac1) for amplifying ~500 bp region of 16S rRNA gene. Sequence runs were submitted to Sequence Read Archive (SRA, National Center for Biotechnology Information, NCBI) under the accession number: SRP022083.

All eukaryotic 454 sequence data devoid of chimeras and poor quality sequences provided by RTL were processed at BioPortal using CLOTU, a pipeline for analysing and processing raw sequence data [11,12]. Any sequences with Ns and shorter than 150 nt in length were excluded. Identical sequences were collapsed and clustering was performed using CD-HIT [13]; CD-HIT parameters: sequence coverage >0.50, sequence identity >0.97, global previse alignment. The resulting clusters were used as queries for blastn against NCBInr database, maintained and updated on the Bioportal [11]. During blast parsing hits not meeting the following criteria were excluded (no-hit/unknown): blastn hit with an overlap more than 50% and with similarity more than 50% [11]. CD-HIT clusters including singletons were imported and aligned with each other in CLC Main Workbench 6.7 (CLC bio, Denmark). The alignments were manually inspected for the presence of possible chimeras and no chimera sequences were detected for Euk1, Euk2 and Euk3 assay clusters. Bacterial sequences were clustered based on 96.5% identity using USEARCH [14]. Cluster file was queried against a database of high quality sequences derived from NCBI utilizes BLASTN+ (KrakenBLAST, www.krakenblast.com) at the RTL and classified at the appropriate taxonomic levels based upon the following criteria. Sequences with identity scores to well characterized 16S rRNA gene sequences, greater than 97% identity (<3% divergence), were resolved at the species level, between 95% and 97% at the genus level, between 90% and 95% at the family and between 85% and 90% at the order level, 80 and 85% at the class and 77% to 80% at phyla. Any match below this percent identity was discarded. Each sequence covering less than 75% of query sequence was discarded. Local blast database was constructed and queried (blastn) using representatives of ARL(I-VIII) clades (see [15,16]) for the presence of eukaryotic chloroplast/plastid small-subunit rRNA gene sequences.

Rarefaction curves, dominance and Chao-1 were calculated in PAST v2.17 [17]. Dominance was calculated using 1-Simpson index of ‘evenness’ and the value ranges from 0 (all taxa are equally present) to 1 (one taxon dominates the community completely). Chao-1 is an estimate of total species richness.

### Amplification of apicomplexan type-N from coral

Coral DNA was subjected to previously described PCR amplifying apicomplexan type-N [9]. Primers were synthesized by Macrogen Ltd. (Seoul, Korea) and PCR amplifications were done with MyTaq Red Mix (BioLine, Australia). DNA was added at a concentration of 100 ng into 50 µL PCR mix. Primers were added at a concentration of 0.20 µM each. The PCR was run using the following cycling conditions: 95 °C for 30 s, 52 °C for 30 s, and 72 °C for 30 s for 35 cycles. All reactions were initiated at 95 °C for 5 min and concluded at 72 °C for 5 min. PCRs were amplified in the Eppendorf Mastercycler Personel. All PCRs were run with negative controls (distilled water instead of sample DNA). Resulting products were resolved in 2% (w/v) agarose. PCR amplicons (~850 bp) were cloned into pCR4 plasmid using TA-TOPO (Invitrogen, Australia). Plasmids were bidirectionally sequenced using M13 primers at Macrogen Ltd. (Seoul, Korea). Sequences were assembled, aligned with related sequences and analysed using CLC Main Workbench 6.7 (CLC bio, Denmark) and deposited in GenBank (NCBI) under the Accession Numbers: KC816717 - KC816723. Evolutionary analyses were conducted in MEGA5.1 [18].

### Amplification of almost complete small-subunit rRNA gene using universal primer sets

The small-subunit rRNA gene was amplified using two primer sets; set E1.2 (18S-42F, 18S-1498R) and set E3.4 (18S-82F, 18S-1520R) [19]. The PCR amplification was performed as previously described [20]. Briefly, MyTaq Red Mix (Bioline, Australia) was used with 0.25 µM concentration of each primer. Template DNA was added at 100-200 ng into the final volume of 50 µL PCR mix. PCR was cycled at 53°C annealing temperature for 30 cycles (denaturing at 95°C for 20s and extension at 72°C for 60s) in an Eppendorf Gradient (Eppendorf, Australia). Primers were synthetized by Pro-Oligo (Sigma-Aldrich, Australia). All PCRs were run with negative controls (distilled water). Amplicons were cloned into pCR4 and transformed into TOP10 competent cells (TA-TOPO, Invitrogen, Australia). Colony PCR products using pCR4 vector specific primers was submitted to Macrogen Ltd. (Seoul, Korea) from sequencing using T7 or/and T3 primers annealing to the pCR4 and flanking the cloned inserts. Sequences were assembled, aligned with related sequences and analysed using CLC Main Workbench 6.7 (CLC bio, Denmark) and deposited in GenBank (NCBI) under the Accession Numbers: KC816631 - KC816716.

Sequences were clustered using CD-HIT with default parameters and sequence identity cut-off set to 0.90, 0.95 and 0.97 [13]. For multiple sequences alignment, of representatives of known *Symbiodinium* spp. clades (A-E) with representatives of Dinozoa were retrieved, alignment constructed and maximum likelihood phylogenetic tree inferred in MEGA5.1 [18].

## Results and Discussion

Three overlapping PCR amplicon 454 pyrosequencing assays (Euk1, Euk2, Euk3) were applied to investigate the diversity and community composition of eukaryotic assemblages in common coral species from the southern Great Barrier Reef (Figure 1). Euk1 assay yielded 24,649 good quality sequences ( *A* *. palifera* 42a: 7,869; *M* *. digitata* 60a: 8,746, *P* *. cylindrica* 66-2: 4,352, *S* *. hystrix* 176-2: 3,682) that formed 72 clusters (97% identity) with more than 1 sequence (25, 33, 13 and 25, respectively) and 62 unique sequences (16, 28, 7 and 19, respectively), all were >150 nt in length and devoid of Ns (Table S1). Euk2 assay yielded 15,204 good quality sequences ( *A* *. palifera* 42a: 3,132; *M* *. digitata* 60a: 3,714; *P* *. cylindrica* 66-2: 4,454; *S* *. hystrix* 176-2: 3,904) that formed 82 clusters (33, 27, 17 and 20, respectively) and 46 unique sequences (21, 13, 11 and 9, respectively), all were >150 nt in length and devoit of Ns (Table S2). Euk3 assay yielded 31,002 sequences, 12 were <150 nt in length and 120 comprised Ns and therefore were excluded. Therefore, we used 30,870 good quality sequences from Euk3 assay ( *A* *. palifera* 42a: 7,927; *M* *. digitata* 60a: 7,352; *P* *. cylindrica* 66-2: 11,734; *S* *. hystrix* 176-2: 3,857) that formed 121 clusters (57, 40, 19, 20) and 143 unique sequences (62, 34, 35 and 16, respectively) (Table S3). Rarefaction curves for each 454 assay and sample demonstrated that sampling was beyond the exponential phase with 25 to 219 taxa estimated using Chao1 (Figure S1). To compare individual assays in their abilities to detect major taxonomic groups, we retrieved taxonomic affiliation for all 97% identity clusters (Figure 1A, Table S1-3). All assays detected hexacoral (Cnidaria) and *Symbiodinium* (Dinophycae) in coral DNA, except Euk3 that performed poorly to detect *Symbiodinium* (Figure 1A). Coral *A* *. palifera* 42a and *M* *. digitata* 60a were dominated by *Symbiodinium* sp. sequences (>70%) using Euk1 and Euk2 assays, while in *P* *. cylindrica* 66-2 and *S* *. hystrix* 176-2 were dominated by hexacoral sequences (>70%) in all three assays (Figure 1A). It is corroborated by the Dominance score >0.30 for all Euk assays (Figure S1). Universal SSU rRNA gene primers set (E1.2 and E3.4, see Material and Methods) confirmed presence of *Symbiodinium* spp. clade C with all four studied coral samples known to dominate the Great Barrier Reef coral [1,2,21] (Text S1). In addition to the cnidarian-*Symbiodinium* symbionts we detected a relatively low number of other protist taxa belonging to apicomplexa, chromerida, non-*Symbiodinium* dinoflagellates, ciliates and diatoms. Protists, excluding *Symbiodinium*, represented only 0.2%, 0.3% and 1.1% of Euk1, Euk2 and Euk3 sequences (Figure 1A). The highest number of protist taxa was identified using Euk3 assay: 17 taxa included alveolates and rhodophytes. Each of the Euk1 and Euk2 assays revealed 9 and 7 taxa belonging to protists, respectively (Table S1-3). Such estimate appears relatively low compared to current data on eukaryotic diversity in ocean [22-24]. One of the protists clusters belonging to ciliates was related to an enigmatic endosymbont *Licnophoramacfarlandi* from the giant California sea cucumber ( *Parastichopus* *californicus* ) [25] in *P* *. cylindrica* 66-2 using all three assays. Euk1 and Euk2 detected diatoms, symbiotic ciliate (cf. *Ophrydium*) and endosymbiotic dinoflagelates (cf. *Amphidinium*) [26-28]. In contrast, Euk3 did not detect any diatoms or stramenopiles in the same coral DNA, suggesting that this assay is now suited for these eukaryotic groups.

**Figure 2:**
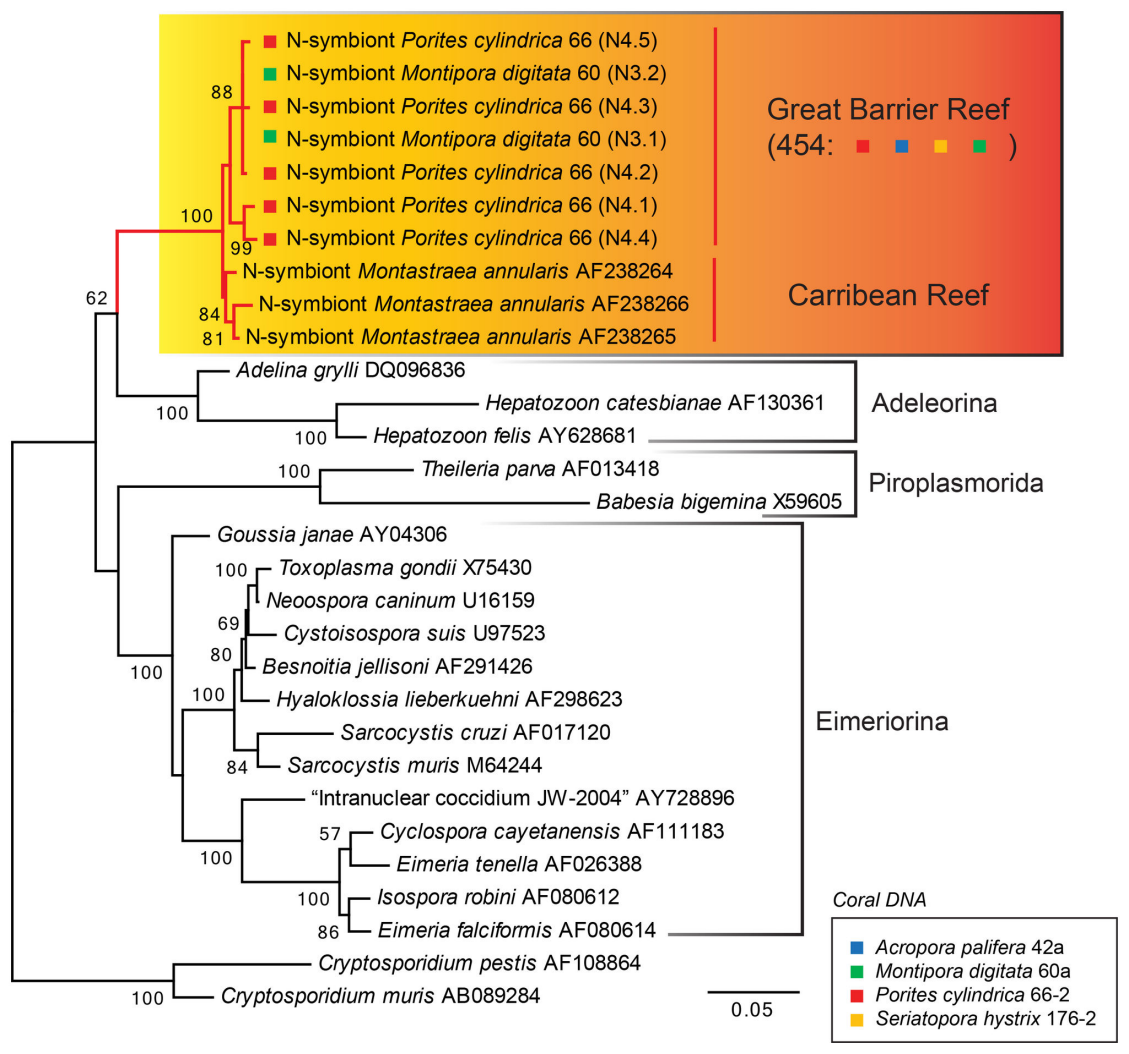
Phylogenetic tree of apicomplexan N-symbiont from the Great Barrier Reef based on small-subunit rRNA gene sequences. The tree was inferred using the Maximum Likelihood method based on the Tamura-Nei model with a discrete Gamma distribution (5 categories, alpha parameter = 0.2444). The tree is drawn to scale, with branch lengths measured in the number of substitutions per site. The analysis involved 30 nucleotide sequences and rooted with *Cryptosporidium* spp. sequences. GenBank accession is shown on the right of the species name. Bootstrap support inferred from 100 replicates is shown (>50%). All positions with less than 75% site coverage were eliminated. There were a total of 1463 positions in the final dataset. Evolutionary analyses were conducted in MEGA5.1.

Using the outcomes of all three 454 assays, we consistently demonstrated the presence of an apicomplexan sequence clusters whose highest BLAST hit was the apicomplexan type-N from a Caribbean coral (AF238264- AF238266) (Table S1-3). An uncorrected p-distance between the apicomplexan nucleotide small-subunit rRNA gene sequence clusters and the apicomplexan type-N sequences was 3-11%, 5-7%, 2-12% based on Euk1-3 assays, respectively (Figure S2). The type-N apicomplexan was first discovered during a coral bleaching experiment by Toller et al. [9] who detected a unique apicomplexan genotype in Caribbean coral and speculated that the sequence may belong to *Gemmocystiscylindrus* [9]. However, the conspecificity of type-N and *G. cylindrus* is pending characterisation of the type-N morphology [9,29]. Using the Euk3 assay, type-N sequence was present in all four examined corals in low abundance (0.02-0.57%). To corroborate our finding using the 454 approach, we subjected coral DNA to PCR [9] that preferentially amplifies apicomplexan type-A PCR amplicon of expected size (~850 bp) was obtained and cloned from two coral DNAs ( *M* *. digitata* 60a, *P* *. cylindrica* 66-2). Sequencing of the cloned inserts confirmed presence of closely related sequences to the apicomplexan type-N from Caribbean coral and polymorphisms detected using 454 (Figure S2). The SSU rRNA gene sequences (818-820 bp) from the Great Barrier Reef coral were 2.1-2.9% distinct (uncorrected p-distance) from the Caribbean type-N sequences. Phylogenetic analysis confirmed monophyly of the type-N sequences within Apicomplexa (Figure 2).

All 454 assays revealed high proportion of unique sequence clusters (77%, 88% and 94% in Euk1-3 assays, respectively; Figure 1B). The sample *M* *. digitata* 60a contained *C* *. velia* sequences using Euk3 (abundance of 0.75%) and a single sequence in Euk2. The sequences of *C* *. velia* were either identical or 0.02% distinct from reference *C* *. velia* strain (JN935829- JN935835 [30]). This finding is supported by recent study that reisolated *C* *. velia* from *M* *. digitata* [31], however, the endosymbiont role of *C* *. velia* within either coral or coral larvae will require further investigation. Interestingly, the sample *M* *. digitata* 60a containing *C* *. velia* revealed presence of two significant human and animal pathogens – the apicomplexan *Cryptosporidium* and the fungus *Cryptococcus* . *Cryptosporidium* spp. are obligate parasites of vertebrates causing profuse diarrhoea [32]. *Cryptosporidium* sequences (abundance 0.7%, Euk3) were either identical to a series of known genotypes of *Cryptosporidium* spp. infecting diverse groups of vertebrates (*C. parvum* sensu lato) or represented a distinct sequence from all previously described *Cryptosporidium* genotypes and species with novel SSU rRNA gene signature [33] (Figure S3). *Cryptosporidium* spp. are waterborne pathogens possessing resilient cyst wall supporting their survival and dispersal in aquatic system with marine bivalves suggested to serve as a reservoir of the infective stages for humans [34]. *Cryptococcus neoformans* and *C* *. gatti* are a serious pathogen of humans that cause respiratory disease [35]. Yet closely related taxa and other members of the genus *Cryptococcus* are reported commonly from sea sediments [36]. The abundance of *Cryptococcus* sp. sequences was 1.2%, 1.5% and 18.3% using Euk1, Euk2 and Euk3 assay, respectively. Comparison of the obtained sequences with a reference alignment of *Cryptococcus* and related Tremellales species revealed that the sequences (Euk1 and Euk2) belong to either *C. neoformans /* *C* *. gatti* and are indistinguishable at the region amplified using either assay (Figure S4). Both *Cryptosporidium* and *Cryptococcus* are animal-associated organisms and their presence may indicate human and animal effluent. Corals were collected in the vicinity of Heron Island which is covered by trees with a large colony of birds, other wildlife, and humans are present on the island all year round. Previously, a enterobacteriaceae ribotype closely related to *Escherichia coli* (γ-proteobacteria) was identified from coral around Heron Island [37]. To confirm the possible presence of effluent, we applied a bacterial PCR amplicon 454 pyrosequencing assays to characterise the bacterial community composition in the same coral DNA samples.

**Figure 3:**
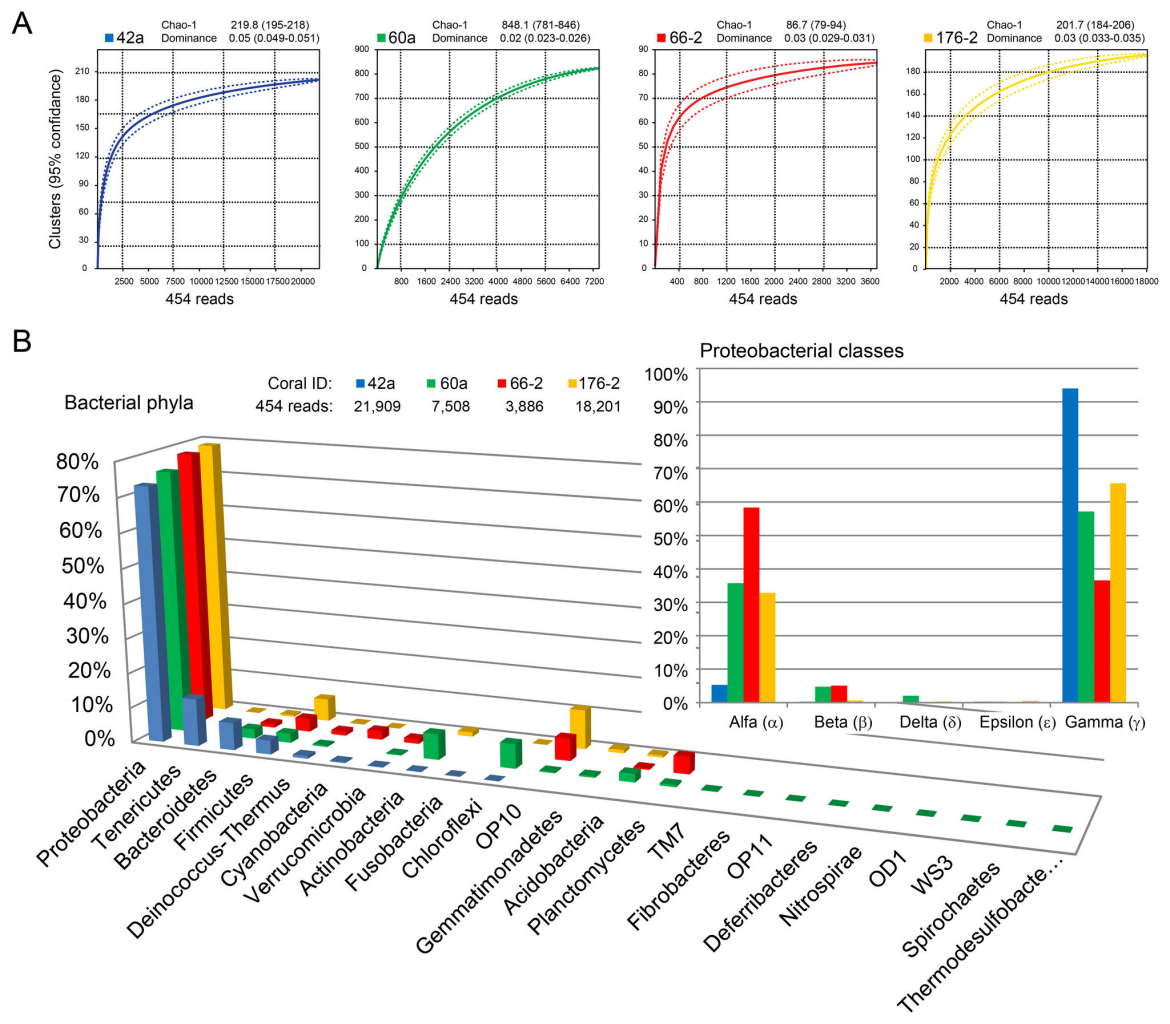
Distribution of bacterial sequences in Great Barrier Reef coral holobionts. (A) Rarefaction curves with 95% confidence interval for sequences obtained using bacterial tag encoded 454 amplicon pyrosequencing assays targeting small-subunit rRNA gene assays (Bac1). Dominance and Chao-1 are indicated above each graph. (B) Abundance of sequences belonging bacterial phyla. Proportion of sequences belonging to bacterial classes within phylum Proteobacteria (inset). Coral species *Acroporapalifera* 42a, *Montipora* *digitata* 60a, *Porites* *cylindrica* 66-2 from Heron Island and *Seriatopora* *hystrix* 176-2 from the One Tree Island.

Proteobacteria sequences dominated coral samples assessed using bacterial PCR amplicon 454 pyrosequencing 16S rRNA gene assay (Bac1). The assay yielded 51,504 good quality sequences ( *A* *. palifera* 42a: 21,909; *M* *. digitata* 60a: 7,508, *P* *. cylindrica* 66-2: 3,886, *S* *. hystrix* 176-2: 18,201) and rarefaction curves demonstrated that sampling was beyond the exponential phase with low (<0.05) dominance score for each sample (Figure 3A). The sample *M* *. digitata* 60a was the most diverse with Chao1 estimate of 848 taxa present compared to the samples with <250 taxa (Figure 3A). Within Proteobacteria, representing 73-79% of sequences within individual coral species, α - and γ-proteobacteria were the most numerous (Figure 3B, inset). However, the ratio between α- and γ-proteobacteria was highly variable ( *A* *. palifera* 42a: 17.8; *M* *. digitata* 60a: 1.6, *P* *. cylindrica* 66-2: 0.6, *S* *. hystrix* 176-2: 2.0). Increase of α-proteobacteria and associated decrease of γ-proteobacteria was documented in reef-building coral in the Caribbean suffering from white plague disease [38]. The least number of α-proteobacterial sequences and the highest α-/γ-proteobacteria ratio was detected in *A* *. palifera* 42a (Figure 3B, inset). At the same time *A* *. palifera* 42a sample did not possess any enterobacterial sequences (γ-proteobacteria), notorious faeces derived pathogens of humans and animals. Importantly, the bacteria of faecal origin (Enterobacteriales) were highly abundant (41%) in the *M* *. digitata* 60a sample accompanied by a moderate α-/γ-proteobacteria ratio in contrast to 0-2% enterobacterial sequences within the coral-associated bacterial communities with and without *C* *. velia* , respectively. Enterobacterial sequences were previously recorded in Heron Island from healthy *Acroporahyacinthus* with a contribution of 21% based on DGGE analysis of 16S rDNA V3 sequence analysis [37]. In this study, sequences classified as belonging to the genus *Escherichia* were recorded in three out of four coral samples ( *M* *. digitata* 60a: 31%, *P* *. cylindrica* 66-2: 2% and *S* *. hystrix* 176-2: 0.05%). Presence of *E. coli* together with *Salmonella* spp. and *Shigella* spp. is the diagnostic indicator of faecal contamination [39,40]. No *Salmonella* sequence was recorded in this study. However, sequences *M* *. digitata* 60a revealed presence of *Shigella* sequences. *Shigella* is mainly associated with humans and primates and therefore indicative of the origin of the contaminating effluent [39,40]. In addition, sequences belonging to the genus *Serratia* were recorded in two coral samples ( *M* *. digitata* 60a, *S* *. hystrix* 176-2) with low abundance of 0.04% and 0.07%, respectively. Several species of the genus *Serratia* are faeces/urine-derived pathogens of humans and animals. Members of the genus *Serratia* have been detected in non-disease coral samples and the genus is thought to be ubiquitous in water [41]. With respect to coral disease one species, *Serratia marcescens*, has been identified as the causal agent of white pox disease in the Caribbean [42]. Using the eukaryotic data above and bacterial Bac1 assay, the *M* *. digitata* 60a sample is considered contaminated by effluent. In addition to the above mentioned bacterial genera ( *Escherichia* and *Shigella* ), the suspected effluent contamination is further supported by the presence of putative waterborne bacterial pathogens, including a low number of sequences belonging to the following genera *Pseudomonas* *,* *Mycobacterium* and *Helicobacter* (Table S4). Both eukaryotic and bacterial assays support effluent exposure of the coral.

Bacterial community assays are known to recover rRNA gene sequences that do not represent extant bacteria but rDNA copies encoded by chloroplast DNA of eukaryotes with chloroplasts or non-photosynthetic plastids [15,16]. Within Bac1 data, we recovered chloroplast/plastid small-subunit rRNA gene belonging to an apicomplexan clade ARL-V clade [15,16] in all 4 coral DNA (abundance 0.3%, 3.7%, 0.2% and 1.0% in sequences from *A* *. palifera* 42a, *M* *. digitata* 60a, *P* *. cylindrical* 66-2 and *S* *. hystrix* 176-2, respectively). The cellular identity of the apicomplexan clade ARL-V is not known despite its ubiquity in our samples. The eukaryotic 454 assay consistently recovered sequences closely related to the apicomplexan N-type. Therefore, it is plausible that the apicomplexan clade ARL-V clade and the apicomplexan type-N clade are derived from the same organism. However, confirmation of this hypothesis will require isolation of the coccidian *G* *. cylindrus* from the Caribbean or related coccidia from the Great Barrier Reef coral.

## Conclusions

Microbial community assessments greatly benefit from cloning-independent and massive parallel approaches facilitated by 454 pyrosequencing technology [43]. The 454 amplicon sequencing technology based on hypervariable region of the small-subunit rRNA gene has been successfully applied to marine protists [22,23], marine picoeukaryotes [24] as well as more targeted surveys (e.g. Perkinsea [44]) and was demonstrated to be superior in detecting rare species, but qualitative diversity outcomes need to be adjusted for confounding effects of rDNA unit copy number variation between different eukaryotes [45,46]. This technology is influenced by selected PCR primers, for example Stoeck et al. [46] have amplified V4 and V9 regions of the small-subunit rRNA gene to detected very different diversity profiles for dinoflagellates within a single DNA sample. Similarly, our results demonstrated that shorter amplicon length assay (<400 bp, Euk1) produce higher richness estimates compared to longer amplicon length assays (>400 bp, Euk2 and Euk 3). The overwhelming dominance of cnidarian and dinoflagellate DNA remains a limitation that was overcome by our sequencing depth for all three assays. Systematic analyses of eukaryotic primer sets, that mimic experimental studies with bacterial primer sets [47], will improve the interpretation and comparison of published microbial diversity datasets.

Nevertheless, baseline data such as molecular diversity is fundamental in disease diagnosis and the understanding of the functional associations and interactions between host, pathogen, and the environment [48]. This study demonstrated the utility of current technologies (e.g. tag encoded 454 amplicon pyrosequencing assays) to document microbial diversity, but at the same time exposed taxon bias when using single small-subunit rRNA gene assays. For coral reef samples, we therefore advocate that a combination of both short and longer assays is applied for a more realistic coral reef microbial eukaryotic diversity survey.

## Supporting Information

Text S1 **Universal SSU rRNA gene primer sets confirm presence of *Symbiodinium* spp. clade C.**
(DOCX)

Figure S1 **Rarefaction curves for Euk1 (A), Euk2 (B) and Euk3 (C) assays with 95% confidence interval.** Coral species *Acroporapalifera* 42a, *Montipora* *digitata* 60a, *Porites* *cylindrica* 66-2 from Heron Island and *Seriatopora* *hystrix* 176-2 from the One Tree Island. Dominance and Chao-1 are indicated above each graph.
(PDF)

Figure S2 **Multiple sequence alignment of small-subunit rRNA gene sequences of the apicomplexan N-type (AF238264- AF238266), sequences obtained using primers that preferentially amplifies apicomplexan type-N** (KC816717-KC816723)** and representative sequences from Euk1-3 assays representing Apicomopexa.**
(PDF)

Figure S3 **Multiple sequence alignment of small-subunit rRNA gene sequences of taxa belonging to parasitic *Cryptosporidium* spp. (Apicomplexa) with sequences from Euk3 assay.** Dominant host is indicated on the left and nucleotide sequence accession number with taxon name on the right.
(PDF)

Figure S4 **Multiple sequence alignment of small-subunit rRNA gene sequences of taxa belonging to saprobic or pathogenic *Cryptococcus* spp. and related fungi (Tremellales).** Sequences from assay Euk3 (upper alignment), Euk2 (middle alignment) and Euk1 (lower alignment). Nucleotide sequence accession number with taxon name on the right.
(PDF)

Table S1 **Summary of Euk1 small subunit rRNA gene 454 assay.**
(XLSX)

Table S2 **Summary of Euk2 small subunit rRNA gene 454 assay.**
(XLSX)

Table S3 **Summary of Euk3 small subunit rRNA gene 454 assay.**
(XLSX)

Table S4 **Classification of bacterial sequences based on Bac1 small subunit rRNA gene 454 assay.**
(TXT)
