# Supplementary material for: Combined Amplicon Pyrosequencing Assays Reveal Presence of the Apicomplexan “type-N” (cf. Gemmocystis cylindrus) and Chromera velia on the Great Barrier Reef, Australia
Source: PLoS One. 2013 Sep 30;8(9):e76095. doi: 10.1371/journal.pone.0076095 (PMC3786883; doi:10.1371/journal.pone.0076095)
Supplement: Text S1 — Universal SSU rRNA gene primer sets confirm presence of Symbiodinium spp. clade C. (DOCX) [file pone.0076095.s001.docx]

**Supplementary text:**

**Universal small subunit rRNA gene primer sets confirm presence of *Symbiodinium* spp. clade C**

Universal small subunit rRNA gene primers set confirmed presence of *Symbiodinium* spp. clade C with all four studied coral samples known to dominate the Great Barrier reef coral [[1-3](#_ENREF_1)]. Two DNA samples were amplified using E1.2 and E3.4 universal small subunit rRNA primers sets amplifying almost complete SSU rDNA. In total, four clone libraries yielded 86 good quality unidirectional sequences of which 27 were sequenced bidirectionally. The *M. digitata* 60a and *P. cylindrical* 66-2 yielded 27 and 25 (E1.2) and 16 and 18 (E3.4) small subunit rRNA gene sequences, respectively. Clustering of all obtained sequences revealed 8, 8 and 7 clusters at 97%, 95% and 90% identity cut-offs, respectively. At the 97% identity cut-off, 6 clusters matched *Symbiodinium* spp. SSU rRNA gene and 2 clusters were related to cnidarian SSU rRNA gene. There was one overlapping cluster of *Symbiodinium* spp. (Cluster 1) amplified from both holobiont total DNAs. There were 7 clusters (6 x *Symbiodinium* spp. SSU rRNA gene and 1x cnidarian small subunit rRNA gene; Clusters 0, 1, 3, 4, 5, 6, 7) from the DNA of *M. digitata* 60a and 2 clusters (1x *Symbiodinium* spp. small subunit rRNA gene and 1x coral small subunit rRNA gene; Clusters 1, 2) from the DNA of *P. cylindrical* 66-2 (Table T1). Multiple sequence alignment identified a range of deletions within the bidirectionally sequenced small subunit rRNA gene of *Symbiodinium* spp. spanning 5, 12, 30, 37, 38, 564 or 804 nt. Phylogenetic analysis of a representative from each of the six clusters belonging to *Symbiodinium* spp. with a selection of known *Symbiodinium* spp. clades (A-E) revealed presence of only clade C in our samples, including representing of the above mentioned truncated forms (Figure T1). This study shows that 454 can be used to assess diversity of *Symbiodinium* spp. clades and would be an appropriate approach for subclade analysis that traditionally employs ITS2 sequencing [[1-3](#_ENREF_1)]. Recently, bacterial 454 assay with real-time PCR for *Symbiodinium* spp. clades C / D, provided detailed evidence that host specificity of bacterial and symbiotic algal communities was insignificant within in Isopora palifera [[4](#_ENREF_4)]. Coupling 454 assay (e.g. *Symbiodinium* specific, bacterilal, eukaryotic) will likely lead to better understanding of emergence and demise of the rare subclades of *Symbuiodinium* spp. within a single coral colony [[3](#_ENREF_3),[4](#_ENREF_4)].

**Table T1: Summary of small subunit rRNA gene survey**

**Figure T1. Phylogenetic tree of *Symbiodinium* spp. based on small subunit rRNA gene sequences.** The tree was inferred using the Maximum Likelihood method based on the Tamura-Nei model with a discrete Gamma distribution (5 categories, alfa parameter = 0.2478). The tree is drawn to scale, with branch lengths measured in the number of substitutions per site. The analysis involved 43 nucleotide sequences (only the *Symbiodinium*-ingroup is shown, outgroup is not shown). GenBank accession or Cluster number is shown for each branch. *Symbiodinium* spp. clade is indicated in the right. All positions with less than 75% site coverage were eliminated. There were a total of 1574 positions in the final dataset. Evolutionary analyses were conducted in MEGA5.1.

**References:**

1. Bongaerts P, Sampayo EM, Bridge TCL, Ridgway T, Vermeulen F, et al. (2011) Symbiodinium diversity in mesophotic coral communities on the Great Barrier Reef: a first assessment. Marine Ecology Progress Series 439: 117-126.

2. Loh WKW, Loi T, Carter D, Hoegh-Guldberg O (2001) Genetic variability of the symbiotic dinoflagellates from the wide ranging coral species Seriatopora hystrix and Acropora longicyathus in the Indo-West Pacific. Marine Ecology-Progress Series 222: 97-107.

3. Stat M, Loh WKW, Hoegh-Guldberg O, Carter DA (2008) Symbiont acquisition strategy drives host-symbiont associations in the southern Great Barrier Reef. Coral Reefs 27: 763-772.

4. Chen CP, Tseng CH, Chen CA, Tang SL (2011) The dynamics of microbial partnerships in the coral Isopora palifera. ISME J 5: 728-740.
