# Supplementary figures and images for: Combined Amplicon Pyrosequencing Assays Reveal Presence of the Apicomplexan “type-N” (cf. Gemmocystis cylindrus) and Chromera velia on the Great Barrier Reef, Australia
Source: PLoS One. 2013 Sep 30;8(9):e76095. doi: 10.1371/journal.pone.0076095 (PMC3786883; doi:10.1371/journal.pone.0076095)

A

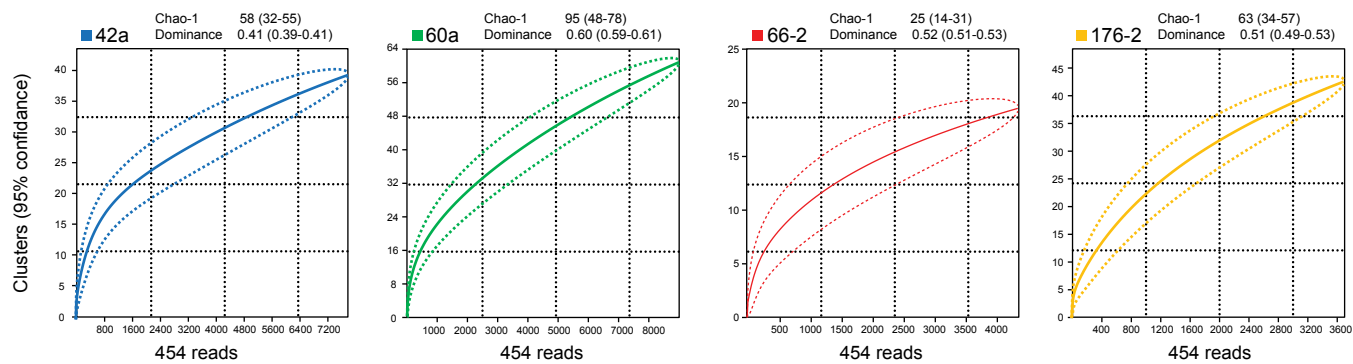

B

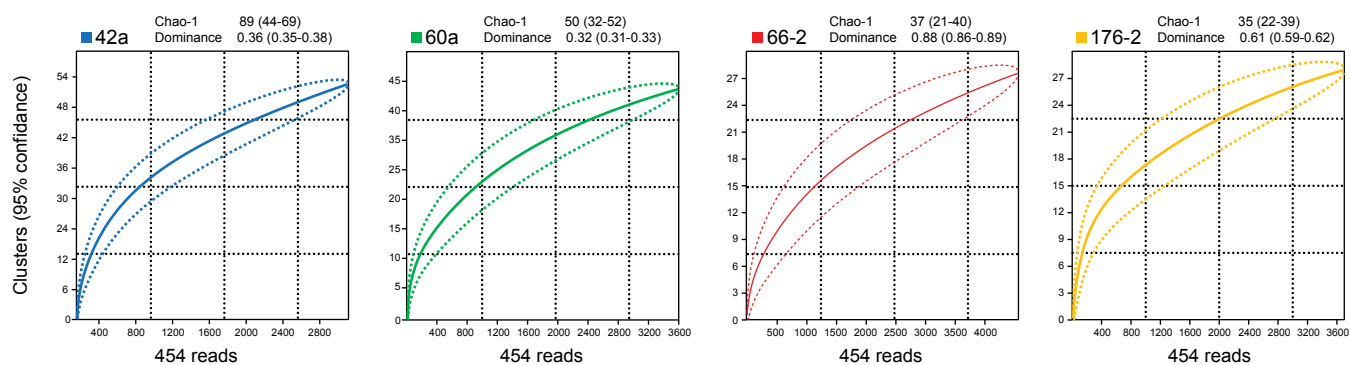

C

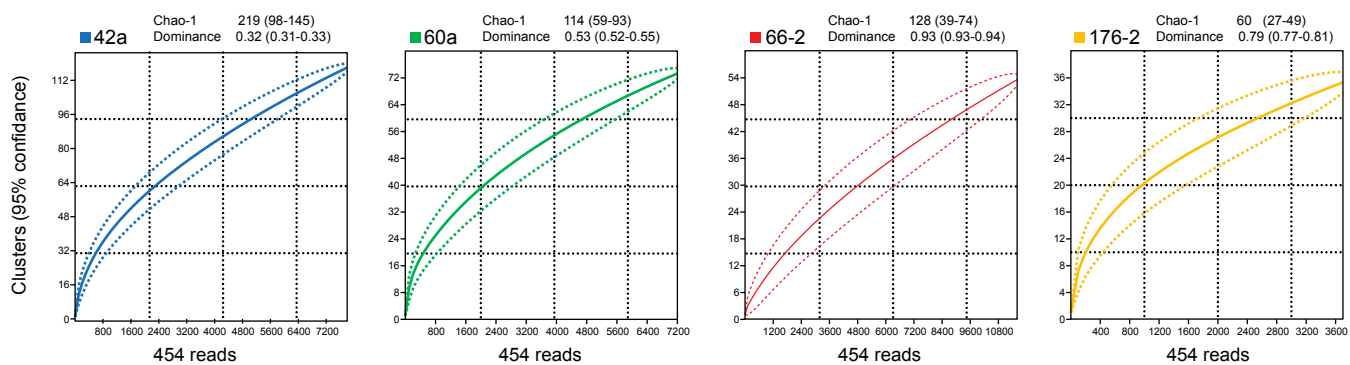

Supplement: Figure S1 — Rarefaction curves for Euk1 (A), Euk2 (B) and Euk3 (C) assays with 95% confidence interval. Coral species Acroporapalifera 42a, Montipora digitata 60a, Porites cylindrica 66-2 from Heron Island and Seriatopora hystrix 176-2 from the One Tree Island. Dominance and Chao-1 are indicated above each graph. (PDF) [file pone.0076095.s002.pdf]

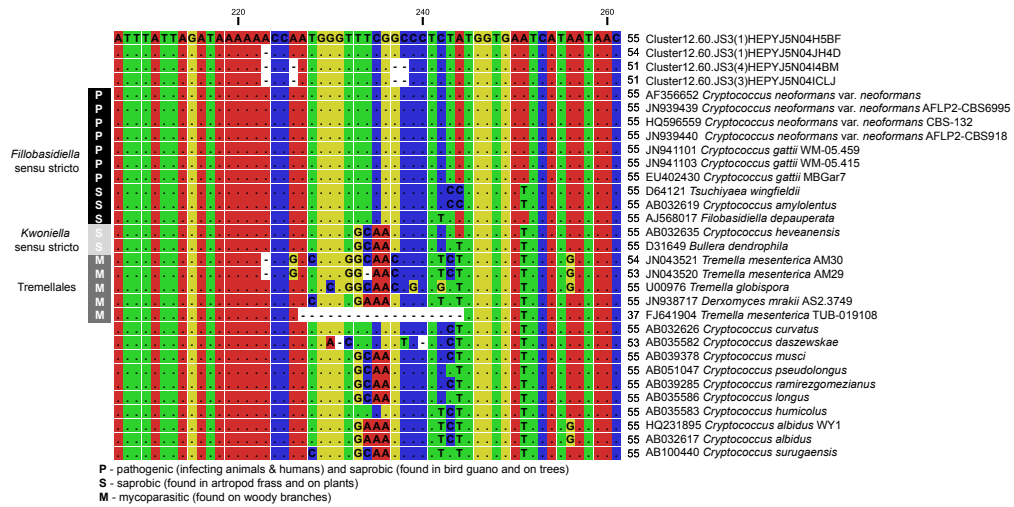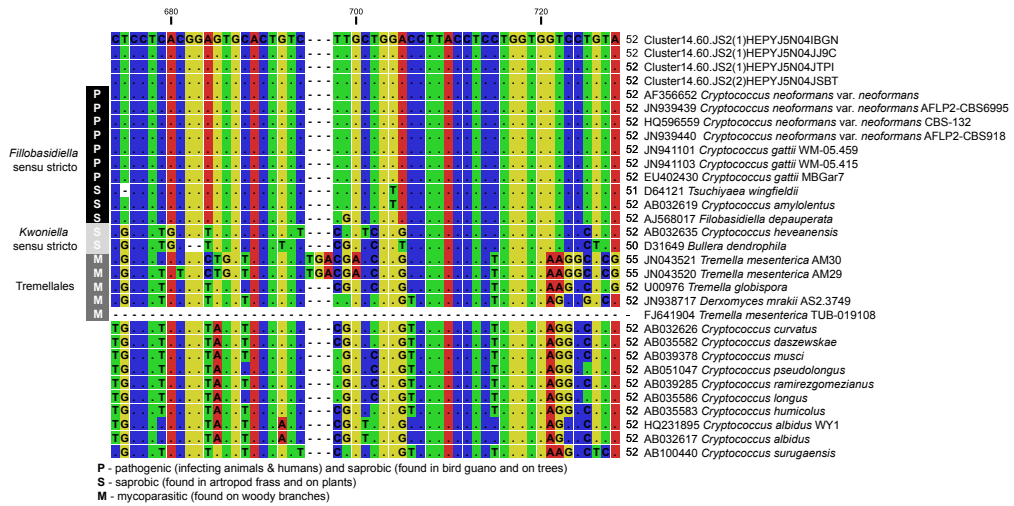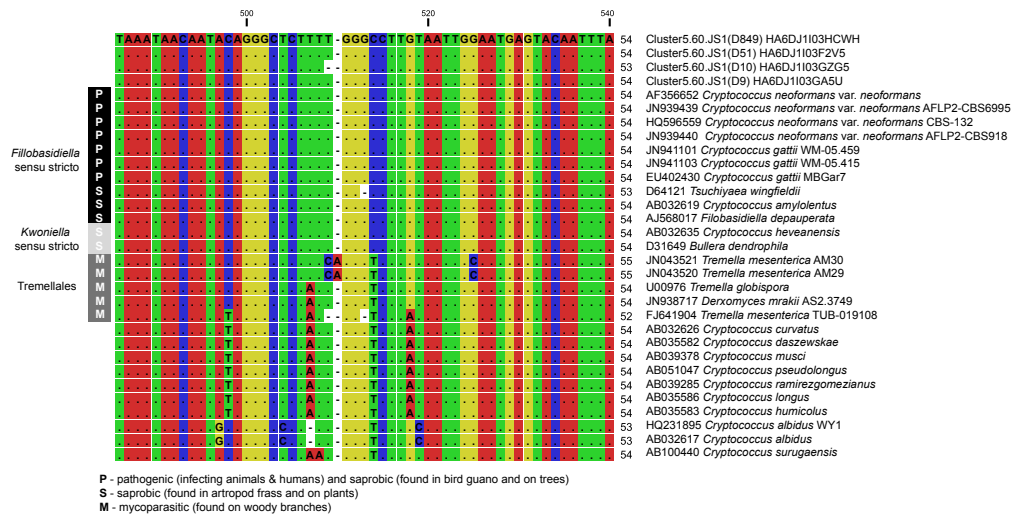

Supplement: Figure S4 — Multiple sequence alignment of small-subunit rRNA gene sequences of taxa belonging to saprobic or pathogenic Cryptococcus spp. and related fungi (Tremellales). Sequences from assay Euk3 (upper alignment), Euk2 (middle alignment) and Euk1 (lower alignment). Nucleotide sequence accession number with taxon name on the right. (PDF) [file pone.0076095.s005.pdf]
