# Supplementary material for: Combined Amplicon Pyrosequencing Assays Reveal Presence of the Apicomplexan “type-N” (cf. Gemmocystis cylindrus) and Chromera velia on the Great Barrier Reef, Australia
Source: PLoS One. 2013 Sep 30;8(9):e76095. doi: 10.1371/journal.pone.0076095 (PMC3786883; doi:10.1371/journal.pone.0076095)
Supplement: Figure S3 — Multiple sequence alignment of small-subunit rRNA gene sequences of taxa belonging to parasitic Cryptosporidium spp. (Apicomplexa) with sequences from Euk3 assay. Dominant host is indicated on the left and nucleotide sequence accession number with taxon name on the right. (PDF) [file pone.0076095.s004.pdf]

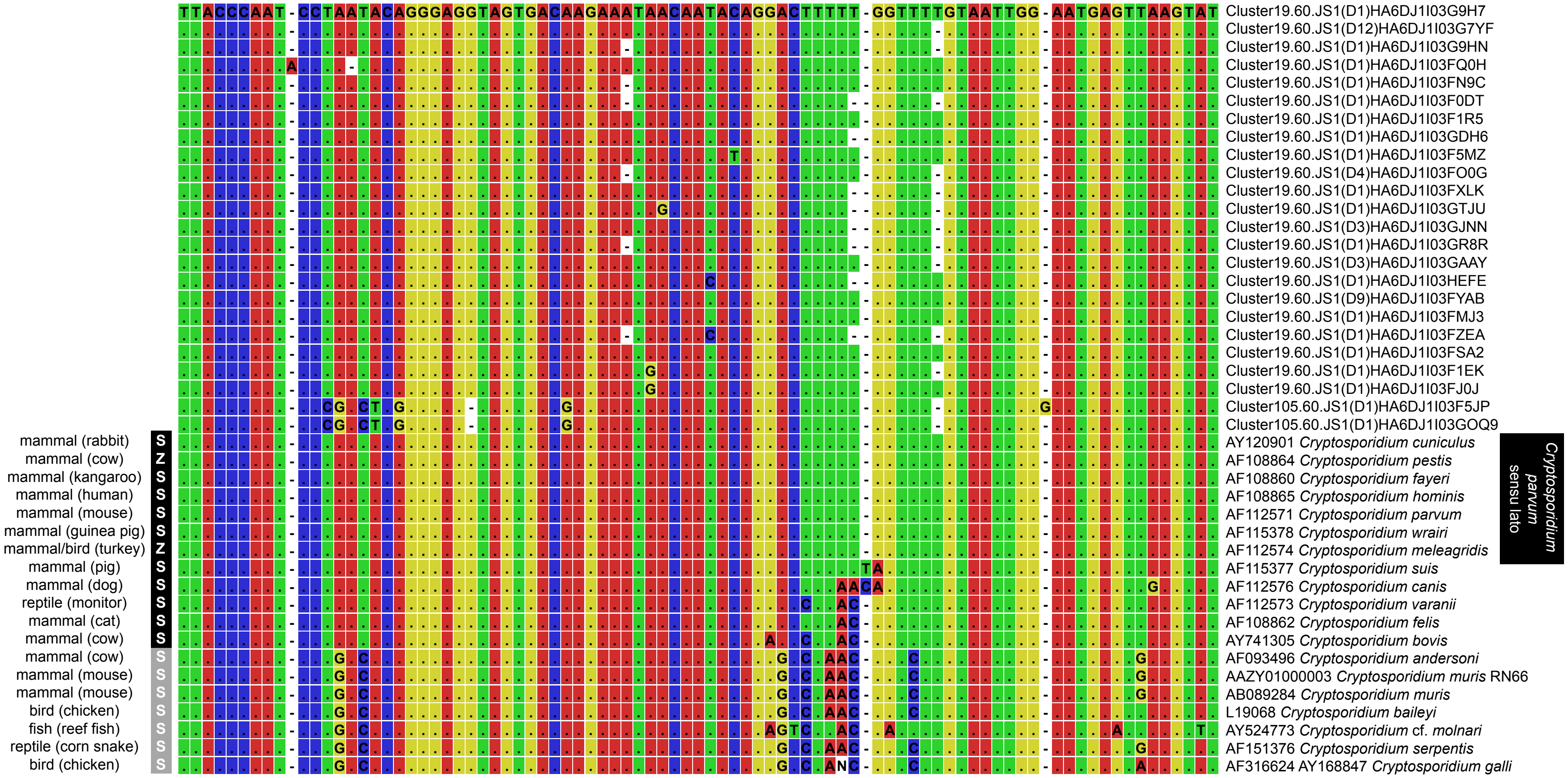

Z - zoonotic (infecting animals & humans)
S - specific (infecting animals, usually a narrow group of hosts)
