# Supplementary material for: Combined Amplicon Pyrosequencing Assays Reveal Presence of the Apicomplexan “type-N” (cf. Gemmocystis cylindrus) and Chromera velia on the Great Barrier Reef, Australia
Source: PLoS One. 2013 Sep 30;8(9):e76095. doi: 10.1371/journal.pone.0076095 (PMC3786883; doi:10.1371/journal.pone.0076095)
Supplement: Figure S2 — Multiple sequence alignment of small-subunit rRNA gene sequences of the apicomplexan N-type (AF238264- AF238266), sequences obtained using primers that preferentially amplifies apicomplexan type-N (KC816717-KC816723) and representative sequences from Euk1-3 assays representing Apicomopexa. (PDF) [file pone.0076095.s003.pdf]

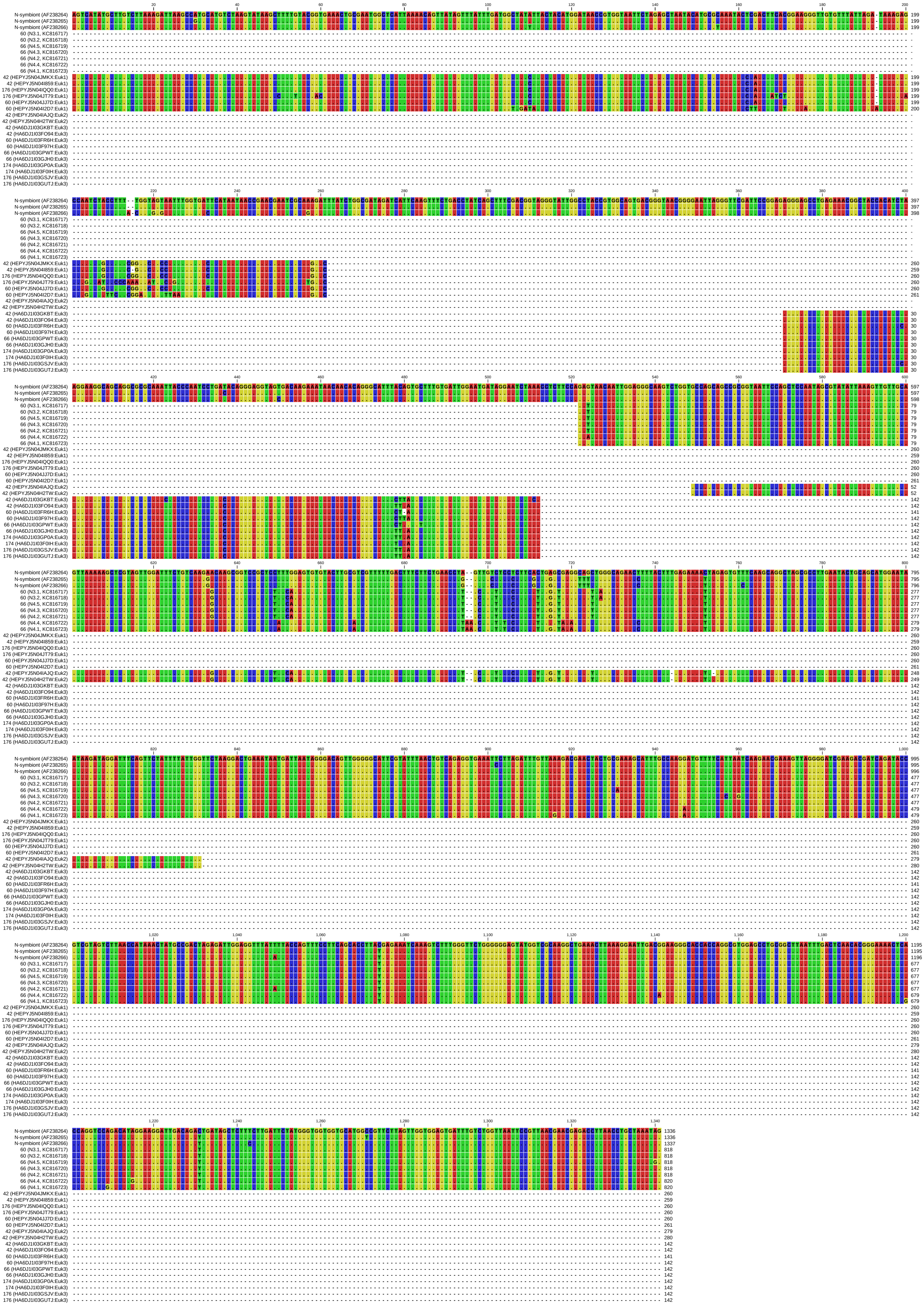

N-symbiont (AF238264)
N-symbiont (AF238265)
N-symbiont (AF238266)
60 (N3.1, KC816717)
60 (N3.2, KC816718)
66 (N4.5, KC816719)
66 (N4.3, KC816720)
66 (N4.2, KC816721)
66 (N4.4, KC816722)
66 (N4.1, KC816723)
42 (HEPYJ5N04JMKX:Euk1)
42 (HEPYJ5N04I859:Euk1)
176 (HEPYJ5N04IQQ0:Euk1)
176 (HEPYJ5N04JT79:Euk1)
60 (HEPYJ5N04JJ7D:Euk1)
60 (HEPYJ5N04I2D7:Euk1)
42 (HEPYJ5N04IAJQ:Euk2)
42 (HEPYJ5N04H2TW:Euk2)
42 (HA6DJ1I03GKBT:Euk3)
42 (HA6DJ1I03FO94:Euk3)
60 (HA6DJ1I03FR6H:Euk3)
60 (HA6DJ1I03F97H:Euk3)
66 (HA6DJ1I03GPWT:Euk3)
66 (HA6DJ1I03GJH0:Euk3)
174 (HA6DJ1I03GP0A:Euk3)
174 (HA6DJ1I03F0IH:Euk3)
176 (HA6DJ1I03GSJV:Euk3)
176 (HA6DJ1I03GUTJ:Euk3)
